# Coral Larvae under Ocean Acidification: Survival, Metabolism, and Metamorphosis

**DOI:** 10.1371/journal.pone.0014521

**Published:** 2011-01-17

**Authors:** Masako Nakamura, Shun Ohki, Atsushi Suzuki, Kazuhiko Sakai

**Affiliations:** 1 Sesoko Station, Tropical Biosphere Research Center, University of the Ryukyus, Okinawa, Japan; 2 Geological Survey of Japan, National Institute of Advanced Industrial Science and Technology (AIST), Tsukuba, Japan; University of California San Diego, United States of America

## Abstract

Ocean acidification may negatively impact the early life stages of some marine invertebrates including corals. Although reduced growth of juvenile corals in acidified seawater has been reported, coral larvae have been reported to demonstrate some level of tolerance to reduced pH. We hypothesize that the observed tolerance of coral larvae to low pH may be partly explained by reduced metabolic rates in acidified seawater because both calcifying and non-calcifying marine invertebrates could show metabolic depression under reduced pH in order to enhance their survival. In this study, after 3-d and 7-d exposure to three different pH levels (8.0, 7.6, and 7.3), we found that the oxygen consumption of *Acropora digitifera* larvae tended to be suppressed with reduced pH, although a statistically significant difference was not observed between pH conditions. Larval metamorphosis was also observed, confirming that successful recruitment is impaired when metamorphosis is disrupted, despite larval survival. Results also showed that the metamorphosis rate significantly decreased under acidified seawater conditions after both short (2 h) and long (7 d) term exposure. These results imply that acidified seawater impacts larval physiology, suggesting that suppressed metabolism and metamorphosis may alter the dispersal potential of larvae and subsequently reduce the resilience of coral communities in the near future as the ocean pH decreases.

## Introduction

Ocean acidification, resulting from an increase in the concentration of atmospheric carbon dioxide (CO_2_) and subsequent increases in the amount of CO_2_ dissolved in oceans, may soon threaten marine calcifying organisms including reef-building corals [1]–[2]. About one quarter of the anthropogenic CO_2_ has been absorbed by oceans over the industrial era [3]–[4]. As a result, ocean pH has already dropped by 0.1 from the pre-industrial era, and it is predicted to further decline by an approximately an additional 0.3 units by the year 2100 [5]–[6]. The conditions of a reduced pH have been reported to cause decreased calcification rates and decalcification in scleractinian corals [7], [8], which are the fundamental organisms in coral reef ecosystems, functioning as reef builders, hosts for primary production by symbiotic zooxanthellae, and habitat providers. However, other than calcification, physio-ecological responses of corals to ocean acidification remain largely unknown. Particularly, the effects of acidification on the early life stages of corals remain largely unexplored, although early life stages of other marine calcifying organisms have been reported to be vulnerable to acidified seawater [9]–[12].

Recently, some studies have focused on the effects of ocean acidification on the early life stages of corals [9], [13]–[16]. These studies have reported that acidified seawater has negative influences on the early life stages of corals, including polyp growth [9], [13], [16] and the establishment of symbiosis between corals and zooxanthellae [16]. However, planktonic coral larvae seem to be tolerant to acidified seawater [16].

Some calcifying and non-calcifying marine invertebrates demonstrate metabolic depression under reduced pH conditions [17]–[18]. This physiological response is often explained as a strategy for enhancing the survival rate of animals under stressful conditions [19]. Therefore, the finding that coral larval survivorship did not significantly differ among different pH conditions [16] may be partly explained by reductions in metabolic rate in acidified seawater.

However, although a reduced pH may not conspicuously inhibit the survival of planktonic coral larvae [9], [13], [16], the growth of juvenile corals after metamorphosis from planktonic larvae may be impacted by acidified seawater as endoskeleton synthesis is required for growth. Metamorphosis is a process that mediates the planktonic larval stage and the benthic juvenile stage; if metamorphosis is disrupted, successful recruitment is impaired, even though the larvae may demonstrate a high survival rate.

The present study first examined the metabolism of coral larvae under different pH conditions to explain the observed, uniform, survival rate of coral larvae under different pH conditions in comparison to a previous study [16]. Therefore, the pH conditions used in the present study followed those in Suwa et al. [16]. We also observed larval metamorphosis after both short (2 h) and long (7 d) term exposure to reduced pH conditions. The metamorphosis processes involve tissue differentiation mediated by differential gene expression and protein synthesis [20]–[23]. The processes are therefore considered to consume significant amounts of energy [24]–[27]. If negative effects on metamorphosis are observed, these may be caused by a lack of energy for metamorphosis and/or by some impact of acidified seawater on the metamorphosis processes. If the metamorphosis rate in acidified seawater decreases after short-term exposure, then the metamorphosis processes are more likely to be impacted by acidified seawater, and it can be concluded that suppressed metamorphosis is not only the result of an energy shortage. In this study, we used larvae of *Acropora digitifera*, which is a common species around Okinawan coral reefs and is also the most commonly used species in studies on the effects of acidified seawater on several early life stages in coral[15]–[16]. As mentioned above, the three pH conditions used in Suwa et al. [16] were employed in the present study; pH 8.0 (control), pH 7.6, and pH 7.3, based on the total hydrogen ion concentration pH scale.

## Materials and Methods

This study was conducted at Sesoko Station, Tropical Biosphere Research Center, University of the Ryukyus, Japan. All samples were collected in strict accordance with good animal practice as defined by the relevant national and/or local animal welfare bodies. All sampling within Okinawa prefecture needing permission for this study was also approved by the Okinawa prefecture, Japan (Permission No. 21–22).

### Larval culture

Sexually mature *Acropora digitifera* colonies were collected from a fringing reef around Sesoko Island, Okinawa Island, Japan (26°38′N, 127°53′E) one week before their predicted spawning date in June 2009. Colonies were kept in a running seawater tank under natural light conditions at Sesoko Station, Tropical Biosphere Research Center, University of the Ryukyus. During *A. digitifera* spawning in June 2009, gametes were collected according to Morita et al. [28]. Planular larvae were obtained by mixing gametes from six spawned colonies in filtered seawater (FSW, pore size 1 µm) at normal pH. The larvae were kept in a container filled with FSW at normal pH by exchanging seawater twice per day to keep the larvae healthy until the initiation of the experiment. Five days post-fertilization, planula larvae were put into bottles or cups for the following experiments (see below).

The pH-adjusted seawater was supplied into each bottle or cup in a flow-through aquarium. The pH-stat system used in this study was the same as that used in Suwa et al. [16]. Seawater, filtered by an inline filter system (0.45 µm), was bubbled with pure CO_2_ to adjust the pH levels of the seawater with a pH regulator, connecting to a pH electrode (Micro-pH; Aquabase, Kanagawa, Japan), and pH levels were adjusted when pH increased to 0.01 higher than the desired levels by injecting pure CO_2_ from the compressed CO_2_ tanks. A modification was made in aquarium replicates from Suwa et al. [16] (three aquaria were used in the present study). The pH conditions of seawater were adjusted to three levels (pH 7.3, 7.6, and 8.0) based on the total hydrogen ion concentration pH scale as in Suwa et al. [16]. Three pH values were theoretically-predicted for the next 300 years (pH 7.3) [29]. These values were chosen to estimate the mechanism of the previously-observed uniformal survival rate of coral larvae under these pH conditions in terms of metabolism, and to investigate the metamorphosis behavior of larvae under acidified conditions. This is because completion of metamorphosis by the surviving larvae is necessary for successful recruitment and survival in acidified seawater. The chemical and physical conditions of each treatment are summarized in Table 1. The pH and temperature were measured every two days, and the mean salinity was measured weekly. The aragonite saturation state was estimated from these parameters by using the computer program CO2SYS [30], using the total alkalinity of 2234 µ mol kg^−1^ reported for Sesoko Island during the experiment. The total alkalinity of the seawater used in the experiment was measured before the experiment. In addition, the total alkalinity was quite stable during the day (unpublished data).

**Table 1:** Summary of physical and chemical conditions in each experimental aquarium.

apH_sws_	a°C	HCO_3_ µmol/kg	CO_3_ µmol/kg	pCO_2_ µatm	Ω_arag_
8.05±0.05	26.3±0.4	1721–1826	208–231	331–397	3.3–3.7
7.57±0.05	26.2±0.4	2002–2057	72–93	1172–1683	1.2–1.5
7.33±0.05	26.6±0.5	2080–2131	42–63	2011–3100	0.7–1.0

The carbon parameters were calculated based on pH_sws_, temperature, salinity 34.0 and a total alkalinity of 2234 µ mol/kg. Ω_Arag_, aragonite saturation state.

a Mean±SD, N = 12.

To investigate survivorship and to measure the oxygen consumption of coral larvae, one hundred planulae were put into a 300-ml bottle, whose opening was covered by a plankton-net (pore size 180 µm) for seawater circulation. The bottle was kept under different pH conditions for 7 days to investigate survivorship and to measure the oxygen consumption of coral larvae. Four bottles were prepared in each flow-through aquarium (N = 12 per pH treatment; 4 bottles per aquarium ×3 aquaria per pH treatment).

To investigate metamorphosis, fifty planulae were maintained in a 230-ml plastic cup, which was covered by a plankton net (pore size 180 µm). Three cups were prepared in each flow-through aquarium (N = 9 per pH treatment; 3 cups per aquarium ×3 aquaria per pH treatment).

### Survivorship and oxygen consumption measurements

**Figure 1:**
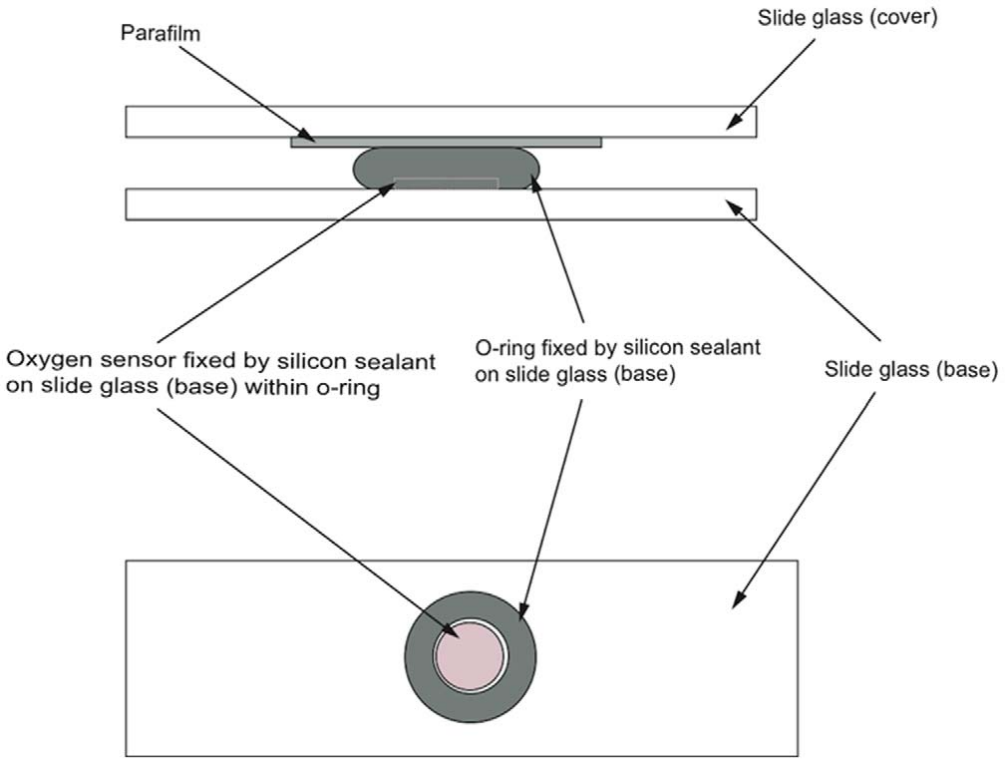
Chamber used for oxygen consumption measurements. Upper, lateral view; lower, upper view.

Larval survivorship and oxygen consumption were observed 3 and 7 days after the initiation of the experiment. Larval survivorship was examined by counting the surviving larvae according to Suwa et al. [16]. Oxygen consumption was measured using the optical O_2_-measuring system (Fibox3, PreSens). Larvae were randomly removed from the bottles, and washed with filtered seawater (pore size 0.22 µm). Twenty larvae were placed into the original 18-µl chamber (Fig. 1). The chamber was placed in a water bath maintained at 27°C by a thermostat (ET-30B, Kotobuki, Osaka, Japan) and an electrical heater (Kotobuki, Osaka, Japan). The temperature was monitored using a digital thermometer (SK-250WP, Sato, Tokyo, Japan) and the oxygen concentration was measured in 10-minute intervals. Values from the fifth to the tenth minute, during which oxygen consumption was stable, were calculated and used as the respiration rate of the larvae as µmoles planula^−1^ h^−1^ at 27°C. After measurement, planulae were returned to the bottles. Five replicates were taken for each pH condition (pH 8.0, 7.6, and 7.3) during each observation period. Thereafter, oxygen consumptions were measured five times, 20 different larvae each time, taken from the three aquaria at each pH condition. The initial oxygen saturation was close to constant among all pH conditions (175–186 µmol/L). The oxygen consumption of larvae after 3 days (Day 3) and 7 days (Day 7) relative to that measured before the initiation of the experiment (Day 0) was calculated.

### Metamorphosis experiment

The ability of the coral larvae to metamorphosize was examined using the coral metamorphosis-inducer peptide Hym-248 [31] after 2 h and 7 d exposure to acidified seawater. Two µl of peptide solution (1×10^−4^ M, dissolved in filtered seawater (FSW2: pore size 0.22 µm)) was added to a well of a 24-well plastic culture plate (3.29 ml per well). A larva kept in acidified seawater was added to 38 µl of FSW and 2 µl of the peptide solution in each well (final peptide concentration: 1×10^−6^ M). Each plate had six wells in a row, and larvae kept under each pH level were added to a row of nine plates. Thus, the completion of metamorphosis was observed for 54 larvae (6 larvae per plate ×9 plates) for each pH condition. The number of metamorphosed larvae was counted 12 h after the addition of the peptide. Larvae were considered to have metamorphosed normally when they became bilaterally symmetric in appearance and stopped rotating at the bottom of the wells.

### Analysis

A partial nested ANOVA was used for analyzing differences in larval survivorship among pH conditions between days. Tukey's HSD multiple comparison tests were used as *post-hoc* tests when the ANOVA detected significant differences. The rate of oxygen consumption relative to initial values observed before the experiment was analyzed by a two-factor ANOVA among pH conditions between observed days. O_2_ consumption was converted into an energy equivalent using the energetic equivalents of Gnaiger [32] (consumption of 1 mole O_2_ = 441.0 kJ mol^−1^). Differences in metamorphosis rates among pH treatments were analyzed by a one-way ANOVA. Data were arcsine-transformed to meet assumptions where necessary.

## Results

**Figure 2:**
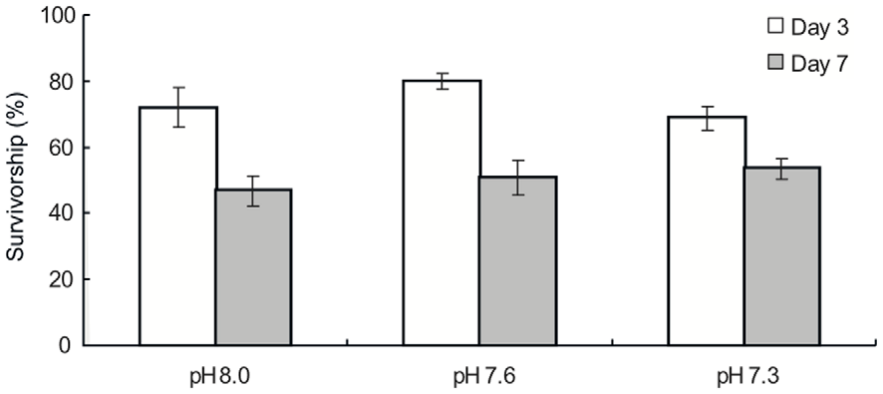
Survivorship of planula larvae of *Acropora digitifera*. Survival rates of the larvae after 3 d (Day 3) and 7 d (Day 7) treatments relative to the number of larvae before initiation of the experiment (Day 0) were compared among three different pH conditions. Bars indicate means and standard errors (*n* = 12).

Larval survivorship differed significantly between observation days, but was not significantly different among pH conditions (Table 2, Fig. 2). A higher number of larvae survived at 3 days (Day 3) than at 7 days (Day 7) after the initiation of the experiment under all pH conditions (Tukey HSD test, *p*<0.001). The survivorship at pH 8.0 was reduced by 25.2% from Day 3 to Day 7. At pH 7.6, survivorship diminished from 79.7±2.3% (mean±S.E.) at Day 3 to 50.6±5.3% at Day 7 (−29.1%). A smaller difference between Day 3 and Day 7 was observed at pH 7.3 (−15.1%).

**Table 2:** Summary of ANOVA results of survivorship of larvae of *Acropora digitifera*.

Source of variation	dF	SS	F	*p*
Day	1	7502.41	43.16	<0.0001
pH	2	360.79	1.04	0.36
Aquarium (pH)	6	750.96	0.72	0.64
Day × pH	2	492.32	1.42	0.25

**Figure 3:**
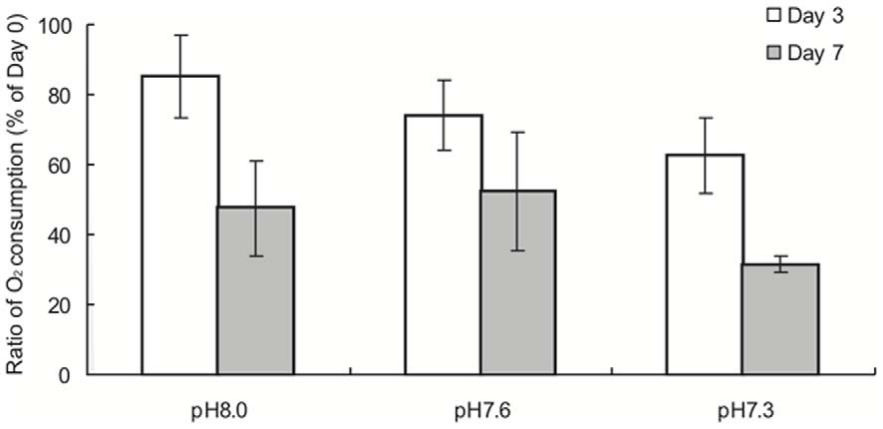
Metabolic rate of planula larvae of *Acropora digitifera*. Oxygen consumption (µmol planula^−1^ h^−1^) of the larvae after 3 d (Day 3) and 7 d (Day 7) relative to that measured before the initiation of theexperiment (Day 0) was compared among three different pH conditions. Bars indicate means and standard errors (*n* = 5).

Oxygen consumption, measured before the experiment (Day 0) and 3 and 7 days after the initiation of the experiment, is summarized in Table 3 with energy equivalents. The ratio of oxygen consumption relative to Day 0 declined significantly from Day 3 to Day 7, but it did not significantly differ among pH conditions (Table 4, Fig. 3) with a power of 0.30 (Least Significant Number  = 10 for each observation). However, the ratio of oxygen consumption decreased as pH decreased at Day 3 (pH 8.0, 84.9±11.7%; pH 7.6, 74.1±10.1%; pH 7.3, 62.4±10.8%). The ratio was similar at pH 8.0 and 7.6, but was lower under pH 7.3 than under other pH conditions at Day 7 (pH 8.0, 47.4±13.5%; pH 7.6, 52.3±17.1%; pH 7.3, 31.5±2.2%).

**Table 3:** Oxygen consumption and converted energy equivalent of planula larvae of *Acropora digitifera*.

	Oxygen consumption [µmol larva^−1^ h^−1^]		(Energy equivalent [J])
Day 0	38.7×10^−5^±5.9×10^−5^		(17.1×10^−5^±2.6×10^−5^)
Day 3	pH 8.0	32.9×10^−5^±4.5×10^−5^	(14.5×10^−5^±2.0×10^−5^)
	pH 7.6	28.6×10^−5^±3.9×10^−5^	(12.6×10^−5^±1.7×10^−5^)
	pH 7.3	24.1×10^−5^±4.2×10^−5^	(10.6×10^−5^±1.8×10^−5^)
Day 7	pH 8.0	18.3×10^−5^±5,2×10^−5^	(8.1×10^−5^±2.3×10^−5^)
	pH 7.6	20.2×10^−5^±6.6×10^−5^	(8.9×10^−5^±2.9×10^−5^)
	pH 7.3	12.2×10^−5^±0.9×10^−5^	(5.4×10^−5^±0.4×10^−5^)

**Table 4:** Summary of ANOVA results of metabolism of larvae of *Acropora digitifera*.

Source of variation	dF	SS	F	*p*
Day	1	6772.94	9.73	<0.01
pH	2	2151.7	1.55	0.23
Day × pH	2	312.62	0.22	0.80

**Figure 4:**
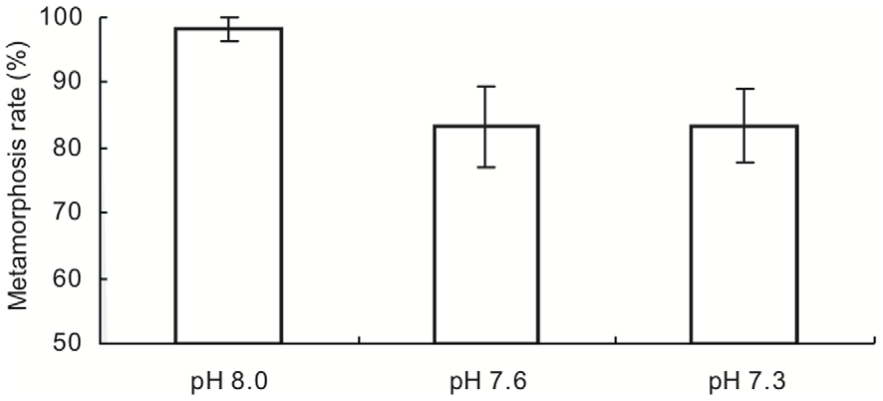
Metamorphosis rate of larvae of *Acropora digitifera* after two-hour exposure to three different pH conditions. Bars indicate means and standard errors (*n* = 9).

The metamorphosis rate was significantly lower under reduced pH conditions than under the control after 2 hours of exposure (Fig. 4). The metamorphosis rate was significantly diminished in acidified seawater (ANOVA, *F_2, 24_* = 3.577, *p* = 0.044). Under control conditions, almost all larvae metamorphosized normally (98.1±1.9%; mean±SE), whereas nearly one fifth of larvae exposed to reduced pH conditions did not metamorphosize completely (pH 7.6, 83.3%±6.2; pH 7.3, 83.3±5.6%).

**Figure 5:**
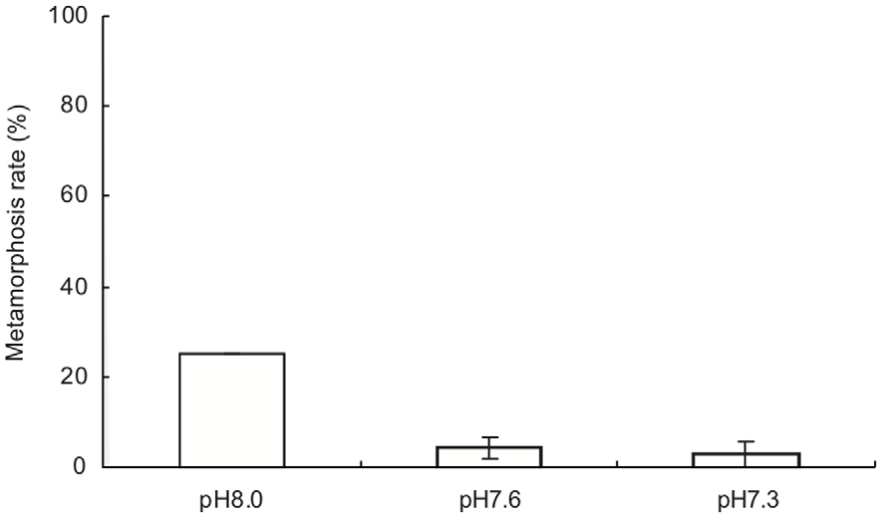
Metamorphosis rate of larvae of *Acropora digitifera* after seven-day exposure to three different pH conditions. Bars indicate means and standard errors (*n* = 9).

Reduced metamorphosis rates were also observed in the 7 d exposure experiment (Fig. 5). The metamorphosis rate of *A. digitifera* significantly decreased with reduced pH (ANOVA, *F_2, 24_* = 29.260, *p*<0.001). It was larger under control conditions than under acidified seawater (pH 8.0, 25.0±0.0%; pH 7.6, 4.2±2.4%; pH 7.3, 2.7±2.7%) (Tukey HSD test, *p*<0.05).

## Discussion

In the present study, although the significant differences were not detected due to the sampling size, the metabolic rates of *Acropora digitifera* larvae, as expressed by oxygen consumption, tended to decrease with reductions in environmental pH during the short term exposure. Metabolic depression with extracellular pH reduction has been similarly reported in other marine invertebrates such as sipunculids (*Sipunculus nudus*, [17]) and mussels (*Mytilus galloprovincialis*, [18]) (reviewed in [33]). Such reduced energy consumption may be related to ion-regulatory processes, which are disturbed by extracellular acidosis [17]. Reipschläger & Pörtner [17] proposed that decreases in environmental pH may induce changes in the ion-exchange transport system for intercellular pH (pH_i_) regulation. Under control conditions, the ubiquitous Na^+^/H^+^ exchanger is actively involved in pH_i_ regulation. However, under reduced pH, it has been reported to be inhibited [34] and to shift to a Na^+^-dependent Cl^−^/HCO_3_^−^ exchanger, such as a Na^+^/H^+^/Cl^−^/HCO_3_^−^ exchanger (e.g. [35]). The latter exchanger is more ATP-efficient and could lead to metabolic depression under low extracellular pH condtions (pH_o_). The above model may apply to coral larvae to explain metabolic depression in relation with pH_o_ changes. Coral reef water, where coral larvae disperse, always shows large diurnal changes in pH (e.g. [36]), and thus coral larvae may have evolved mechanisms similar to those in the above model for regulating ionic balance.

Metabolic suppression was argued to be an energy-saving strategy for survival under stressful conditions over short periods in many organisms [19]. Similar to the previous study [16], the survivorship of coral larvae did not statistically differ among different pH conditions in the present study. In addition, metabolism showed some declining trend with reduced pH in the short term (3 days). These results suggest that coral larvae may lower their metabolic rate in acidified seawater to enhance their survival rate at least in the short term. Similarly, the difference in survivorship between Day 3 and Day 7 was the smallest at the lowest pH (pH 7.3) (Fig. 2) and oxygen consumption was the lowest for both observations (Day 3 and Day 7) (Fig. 3). This may imply that larvae kept in acidified seawater decreased their metabolism in order to survive at least under a low pH condition. However, such metabolic suppression may diminish the survival of coral larvae over long periods. A reduction in energy turnover may have also evolved as a mechanism to withstand temporal changes in environmental CO_2_ (review in [33]). For example, the sipunculid *Spinuculus nudas* showed pronounced sensitivity to CO_2_ changes during long-term exposure, while tolerating temporary exposure by reducing their metabolism [37]. Metabolic suppression may not be advantageous under chronic CO_2_ elevations, as metabolic suppression probably induces reductions in protein synthesis. In addition, the ability to upregulate the mRNA expression of stress proteins under stressful environmental conditions decreased under a reduced pH [38]. Therefore, larvae with lower metabolic rates may be damaged with longer exposure to a reduced pH because some coral larvae potentially have the ability to disperse for more than 100 days [39]. That is, over long periods, coral larval survivorship may be negatively affected by acidified seawater. If this is the case, connectivity among local populations may be disrupted by reductions in the dispersal distance of coral larvae.

However, the decline in the oxygen consumption rate observed in the control treatment with time in the present study may be a natural process. Some acroporid coral larvae previously demonstrated a downward trend in metabolism after 5 days after spawning [40]–[41]. This was demonstrated in *Acropora tenuis*, where the total lipid content of larvae dramatically decreased 5 days after spawning [40]. Over the same time span (5 days after spawning), the peak of oxygen consumption rate was observed for larvae of *A. intermedia* [41]. In addition, the total lipid content and oxygen consumption of both acroporid coral larvae decreased gradually 5 days after spawning [40]–[41]. These results suggest a decline of the metabolism in acroporid coral larvae beginning 5 days after spawning under natural conditions. In our investigation, 5 day-old larvae of *A. digitifera* were used for oxygen consumption analysis. Therefore, the decline of oxygen consumption of the larvae was expected with time even in the control treatment as observed in the present study.

Suppressed metamorphosis rates of *Acropora* spp. larvae after a 2 h exposure to acidified seawater may indicate the possibility of abruption of the metamorphosis process, such as relative gene and protein expression. Metamorphosis entails the reorganization of existing tissues and construction of new tissues, with biochemical and physiological changes mediated by differential gene and protein expressions [20]–[23]. In the metamorphosis of corals, larvae contract in the oral–aboral plane when attached to the appropriate substrata. Then they metamorphose into a flattened disc, followed by the onset of calcification and the formation of calcareous aggregates beneath the flattened aboral epidermis. This transformation involves the secretion or loss of the heterogeneous cell types of the larval epidermis and their replacement by a single calicoblastic cell type [42]. Moreover, gene expression in metamorphosed juveniles largely differs from that in planktonic larvae [43]. Therefore, completion of metamorphosis is potentially energetically costly [23]. As coral larvae are lecithotrophic and use stored lipids as a major energy source [40], it may be difficult to determine whether metamorphosis suppression after 7-d exposure to acidified seawater is caused by energy depletion and/or by abnormalities in metamorphosis. However, the observed reduction in metamorphosis rates of coral larvae that were exposed to acidified seawater for only 2 h implies that acidified seawater abrupts the processes involved in metamorphosis.

The reduced metamorphosis of acroporid coral larvae in acidified seawater suggests potentially harmful effects of acidified seawater on corals. Furthermore, the effects may be underestimated for the following reason. The present study exposed larvae to the metamorphosis inducer Hym-248 according to Iwao et al. [31] rather than using natural inducers such as crustose coralline algae [44]. Hym-248 is one member of the GLWamide peptide family, which is thought to act hormonally to trigger metamorphosis in some hydrozoan and coral species [31], [45]–[46]. This peptide is suggested to mimic endogenous molecules to initiate larval metamorphosis in *Acropora* spp. and to induce metamorphosis with high efficiency, taking a much shorter time to complete metamorphosis compared to metamorphosis induced by CCA [31]. Therefore, the present results may overestimate the metamorphosis rate of larvae under acidified conditions. As such, further elucidation of the effects of acidified seawater on the metamorphosis of coral larvae are required using (1) pH values that are more suitable to the predicted natural changes and (2) natural metamorphosis inducers such as CCA.

The completion of metamorphosis is necessary for successful recruitment as well as larval supply. The metamorphosis of coral larvae is primarily induced by various species of crustose coralline algae (CCA) [44]. Experimental works have demonstrated the negative effects of ocean acidification on CCA [47]–[48]. Kuffner et al. [48] reported that the recruitment rate and percentage cover of CCA was significantly reduced in acidified conditions after seven weeks. In addition, the calcification of the CCA *Porolithon onkodes* was largely reduced under acidified conditions [47]. CCAs consist of Mg calcite, which is more soluble in calcium carbonate than are aragonite and calcite [49]. Therefore, Mg calcite precipitation and deposition of these minerals in organisms may be greatly affected when carbonate saturation states decline due to ongoing acidification. Reduction in CCA due to ocean acidification presumes fewer places available for coral recruitment. Therefore, ocean acidification may have an impact on the sustainability of coral communities by interacting effects of reduced metamorphosis rates and less available CCA onto which corals can recruit.

In summary, our results suggest that as a result of chronic exposure to acidified seawater, coral larvae may be negatively impacted in regards to their survival and metamorphosis processes. However, they apparently show some level of tolerance for short-term exposures to acidified seawater. Therefore, if the emission of anthropogenic CO_2_ is not reduced significantly in the near future and oceans continue to be acidifying, ocean acidification may alter the connectivity between coral populations and reduce their resilience after severe disturbances. This may induce loss of habitat complexity in coral reef ecosystems and subsequent losses in coral reef biodiversity.
